# NADH supplementation improves human oocyte maturation and developmental competence of resulting embryos in controlled ovarian hyperstimulation cycles: a pilot study implicating the CDK2/GAS6 signaling pathway

**DOI:** 10.3389/fendo.2025.1627679

**Published:** 2025-09-03

**Authors:** Qiqi Zhang, Han Yang, Dandan Yang, Hedong Lu, Min Xiong, Lanxin Xie, Yongqi Fan, Kuanjian Zhang, Chao Zhang, Tingting Ye, Ding Ding, Weiwei Zou, Dongmei Ji, Beili Chen, Qiushuang Wang, Huijuan Zou, Zhiguo Zhang

**Affiliations:** ^1^ Department of Obstetrics and Gynecology, the First Affiliated Hospital of Anhui Medical University, Hefei, Anhui, China; ^2^ National Health Commission (NHC) Key Laboratory of Study on Abnormal Gametes and Reproductive Tract, Anhui Medical University, Hefei, Anhui, China; ^3^ Engineering Research Center of Biopreservation and Artificial Organs, Ministry of Education, Hefei, Anhui, China; ^4^ Case Management Department, the First Affiliated Hospital of Anhui Medical University, Hefei, Anhui, China; ^5^ Key Laboratory of Population Health Across Life Cycle, Anhui Medical University, Ministry of Education of the People’s Republic of China, Hefei, Anhui, China; ^6^ Anhui Province Key Laboratory of Reproductive Disorders and Obstetrics and Gynaecology Diseases, Hefei, Anhui, China; ^7^ Biopreservation and Artificial Organs, Anhui Provincial Engineering Research Center, Anhui Medical University, Hefei, Anhui, China; ^8^ Anhui Provincial Institute of Translational Medicine, Hefei, Anhui, China; ^9^ Innovation Research Institute of Engineering Medicine and Medical Equipment of Anhui Medical University, Hefei, Anhui, China

**Keywords:** *in vitro* maturation, nicotinamide adenine dinucleotide, immature human oocyte, maturation, embryonic development

## Abstract

**Background:**

Mitochondrial dysfunction in immature oocytes remains a critical barrier to successful *in vitro* maturation (IVM), particularly in cases of diminished ovarian reserve. While NAD^+^ precursors are extensively studied, the direct impact of NADH - the reduced form central to electron transport - on human oocyte maturation remains unexplored.

**Methods:**

Discarded GV/MⅠ oocytes from controlled ovarian hyperstimulation (COH) cycles were randomized to IVM media supplemented with NADH (10^-8^-10^–4^ M). The optimal concentration (10^–6^ M) was determined by embryonic development. Mechanistic analyses included: mitochondrial phenotyping, single-cell RNA sequencing (scRNA-seq) and intervention experiments.

**Results:**

NADH boosted maturation rates by 26.31% and blastocyst rates by 23.4% versus controls. Mitochondrial indices surged (ATP, mitochondrial membrane potential, glutathione, all P < 0.05), accompanied by ROS reduction. scRNA-seq and immunofluorescence results revealed NADH upregulated CDK2 and GAS6 genes. CDK2 inhibition suppressed maturation (5.13%), while NADH co-treatment partially restored rates (34.21%) after 24 hours. Exogenous GAS6 enhanced blastocyst formation by 44.44%.

**Conclusion:**

This pilot study demonstrates that NADH, as a mitochondrial bioenergetic enhancer, ameliorates Human oocytes maturation and subsequent embryonic development, with this promotive effect appearing to be associated with upregulation of CDK2 and GAS6.

## 1 Introduction

The ovarian follicular reserve, established during fetal development, undergoes progressive depletion through physiological attrition and cyclic recruitment during reproductive lifespan. This finite biological process typically yields approximately 400 mature oocytes throughout a woman’s reproductive career, establishing oocytes as an exceptionally valuable biological resource. In contemporary assisted reproductive technology (ART) practice, controlled ovarian hyperstimulation (COH) cycles yield 10%-15% immature oocytes (germinal vesicle: GV or metaphase I: MI stage) that remain clinically unusable for conventional fertilization protocols (1, 2). Current laboratory protocols typically discard these developmentally compromised gametes due to their limited developmental competence (3, 4). However, for specific patient populations including those with polycystic ovary syndrome (PCOS) (5), oocyte maturation defect (OMD) (6, 7), or premature ovarian failure (POF) (8), these immature oocytes represent critical reproductive potential. Effective utilization of these gametes through advanced culture strategies could substantially improve fertility outcomes in these challenging clinical scenarios. The clinical application of immature oocytes retrieved during COH cycles fundamentally depends on the refinement of *in vitro* maturation (IVM) technology, which currently faces significant limitations in maturation efficiency and developmental consistency.

Contemporary IVM protocols frequently incorporate mitochondrial enhancers and reactive oxygen species (ROS) scavengers, exemplified by melatonin (MT), to optimize cytoplasmic maturation. Our previous investigations demonstrated that mitochondrial bioenergetic augmentation, particularly through enhanced adenosine triphosphate (ATP) biosynthesis capacity, constitutes a critical determinant of IVM success (9). Through systematic modulation of mitochondrial redox homeostasis via MT supplementation, we achieved significant improvements in ATP production and subsequent embryonic developmental potential in IVM-derived oocytes (10–13). Building upon these findings, we propose a novel strategy targeting electron transport chain complex I (ETC CI) function through direct supplementation with reduced nicotinamide adenine dinucleotide (NADH) - the primary electron donor in oxidative phosphorylation. This approach potentially circumvents indirect antioxidant pathways by directly enhancing ETC efficiency.

The NADH/NAD^+^ redox couple serves as a central metabolic node regulating cellular bioenergetics, genomic stability, and redox homeostasis (14–18). Age-associated NAD^+^ depletion has been mechanistically linked to mitochondrial dysfunction, oxidative stress accumulation, and compromised DNA repair mechanisms (19, 20). Emerging evidence positions NAD^+^ metabolism as a critical regulator of oocyte quality, with preclinical studies demonstrating NAD^+^ precursor supplementation (nicotinamide riboside [NR], nicotinamide mononucleotide [NMN], nicotinamide [NAM]) enhances oocyte competence in mouse (21), porcine (22), and bovine (23) models. Notably, Xiong et al. and Bertoldo et al. established that NMN administration rescues age-related oocyte defects through mitochondrial protection, ROS mitigation, and apoptosis suppression in reproductively aged murine models (24, 25). Complementary findings by Brigitte et al. in guinea pig cardiomyocytes demonstrated direct ATP enhancement through NADH supplementation, suggesting potential translatability to oocyte energy metabolism (26). Compared to NAD^+^ precursors requiring enzymatic activation, NADH serves as the direct electron donor for mitochondrial complex I. This bypasses metabolic bottlenecks (e.g., NMNAT-limited NAD^+^ synthesis) to enable rapid bioenergetic rescue. Its dual capacity to drive ATP production while maintaining redox balance provides comprehensive metabolic support for maturation-resistant oocytes (27).

Building upon our prior investigations into IVM methodologies, this study investigated the development and application of an advanced IVM culture system supplemented with NADH for enhancing the maturation competence of human immature oocytes. This investigation aimed to systematically evaluate the biochemical properties, maturation efficacy, and underlying molecular mechanisms of this optimized culture system, while simultaneously establishing foundational knowledge for refining IVM protocols to enable clinical utilization of immature oocytes retrieved during COH cycles.

## 2 Materials and methods

### 2.1 Chemicals and reagents

Chemicals and reagents: NADH and 17β‐Estradiol (E2) were purchased from Sigma-Aldrich (St. Louis, MO, USA). Embryo culture media series (Gamete Buffer™, Fertilization Medium™, Cleavage Medium™, Blastocyst Medium™) were purchased from Cook Medical (Bloomington, IN, USA). Recombinant human FSH (Gonal-f^®^; Merck Serono, Geneva, Switzerland). Human chorionic gonadotropin (Livzon^®^; Livzon Pharmaceutical, Zhuhai, China).

### 2.2 Immature oocyte collection

Discarded oocytes (GV/MI stage oocytes) were obtained from patients undergoing COH cycles treated with intracytoplasmic sperm injection (ICSI) at the Reproductive Medicine Center of the First Affiliated Hospital of Anhui Medical University (August 2021 to June 2024). Exclusion criteria included: donor-derived oocytes; cytogenetically abnormal oocytes (validated by preimplantation genetic testing for aneuploidy); morphologically compromised oocytes (defined by cytoplasmic darkness, > 10% vacuolization, or zona pellucida abnormalities).

### 2.3 Study design

**Figure 1:**
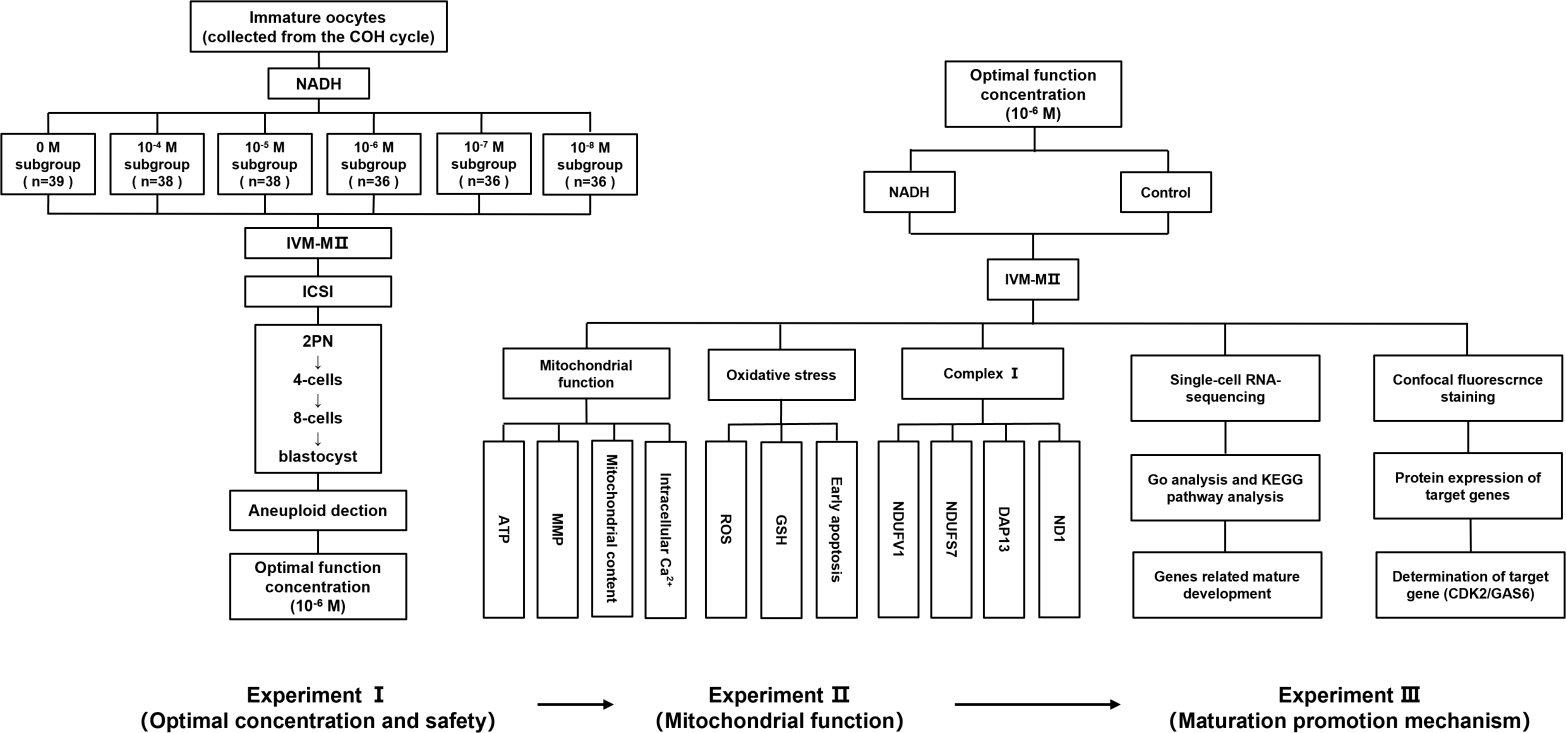
A flow chart of the experimental design.

This research encompasses three experimental phases. In Experiment I, GV/MI oocytes were collected from 119 COH cycle patients (baseline characteristics in Appendix B.1: **Supplementary Table S1**) and allocated into groups with varying NADH concentrations (0, 10^-4^, 10^-5^, 10^-6^, 10^–7^ and 10^–8^ M). IVM for 24 hours in 6% CO_2_/5% O_2_ at 37 °C, the *in vitro*-matured metaphase II IVM-MII) oocytes underwent ICSI. Utilize EmbryoScope^®^ time-lapse system (Vitrolife, Gothenburg, Sweden) for 6 days’ culturing. We evaluated the rates of maturation (GV/MI to MII), fertilization (2PN formation), Embryo quality (Day 3: ≥ 6 cells, < 20% fragmentation; Day 5-6: Gardner grading). Additionally, aneuploidy in high-quality blastocysts was assessed to ensure embryonic safety (More details for “IVM medium preparation”, “IVM, ICSI, and embryo culture”, and “Blastocyst grading” in the **Supplementary Material 1**). The optimal NADH concentration was determined based on the outcomes of early embryonic development and applied into the following experiments. In Experiment II, to investigate the impact of NADH on mitochondrial functional in mature human oocytes *in vitro*, GV/MII oocytes were cultured both with NADH (NADH group) and without NADH (control group). After 24 hours, MII oocytes were collected to evaluate mitochondrial function in the oocytes using laser confocal fluorescence staining. In Experiment III, single-cell sequencing and immunofluorescence were employed to elucidate the mechanisms by which NADH affected human oocyte maturation and embryonic development. And use the inhibitor/protein addition experiment of key molecules to further verify the experimental findings. The experimental flow chart is illustrated in **Figure 1**.

### 2.4 Examination of aneuploidy of blastocyst

The high-quality blastocysts formed in Experiment I were selected and the aneuploidy of blastocysts was detected by array CGH method. (For more details, see the **Supplementary Material 1** “Array CGH Protocol”).

### 2.5 ATP level quantification

MII oocytes were washed three times in pre-warmed PBS (37 °C) and fixed in 4% paraformaldehyde (PFA) for 1 hour under light-protected conditions. Following PBS rinses, oocytes were incubated with 500 nM ATP-sensitive fluorescent probe (ATP Determination Kit, Invitrogen) in PBS at room temperature for 1 hour. After staining, oocytes were transferred to glass-bottom dishes and imaged using a Zeiss LSM 880 confocal microscope. Fluorescence intensity was quantified with identical acquisition parameters (488 nm excitation). At least three biologically independent COH cycles were included and three repeated experiments were performed.

### 2.6 Mitochondrial mass assessment

Mitochondrial content was evaluated using MitoTracker^®^ Green FM (Invitrogen). Oocytes were incubated with 100 nM dye in PBS at 37 °C under 5% CO_2_ for 45 minutes, followed by three PBS washes. Confocal imaging was performed under standardized settings, with fluorescence intensity quantified relative to negative controls. Data obtained from at least three independent COH cycles with three experimental replicates.

### 2.7 Multiparametric redox status analysis

Mitochondrial membrane potential (MMP) was assessed via JC-1 staining (4 μL/ml, Beyotime). Oocytes were incubated for 20 minutes at 37 °C, with fluorescence ratios calculated to determine polarized/depolarized mitochondria. Intracellular calcium (Fluo-4 AM; 5 μM), reactive oxygen species (DCFH-DA; 10 μM), and glutathione (GSH) (CellTracker™ Blue; 10 μM) levels were concurrently measured using spectrally distinct probes. Following 30-minute incubations, oocytes were imaged sequentially using predefined filter sets. ImageJ was employed for background subtraction and intensity quantification, with three technical replicates per experimental condition (at least three biologically independent COH cycles).

### 2.8 Assessment of early apoptosis in oocytes

Oocytes were washed three times in 0.1% polyvinyl alcohol (PVA)/PBS and stained with Annexin-V-FITC (Vazyme, Nanjing, China) in binding buffer for 10 minutes at 37 °C. Confocal imaging (Zeiss LSM 880) distinguished apoptotic (oolemma + zona pellucida fluorescence) from viable (zona-only signal) oocytes. The percentage of oocytes exhibiting early apoptosis was quantified, with three independent experimental replicates being performed (including at least three COH cycles).

### 2.9 Single-cell RNA sequencing analysis

Oocytes (n = 6 per experimental group) were prepared for scRNA-seq analysis with triplicate biological replicates. All specimens were cryopreserved at -80 °C in RNAlater stabilization solution (Beijing Genomics Institution, Shenzhen, China) until processing. RNA extraction was performed using a RNeasy Plus Mini Kit (Qiagen, Germany) according to the manufacturer’s specifications. Detailed procedures are provided in **Supplementary Material 1**.

### 2.10 Immunofluorescence detection of proteins expression

The anti-NDUFS7 antibody, anti-NDUFV1 antibody, anti-ND1 antibody and anti-DAP13 antibody were purchased from Wuhan Fine Biotech Co., Ltd (Wuhan, China). The CDK2/GAS6/DMC1 antibodies were purchased from Affinity Bioscience Co., Ltd (Jiangsu, China). FAM9B Rabbit pAb was purchased from Beijing Bioss Co., Ltd (Beijing, China). C14orf39 Polyclonal antibody was purchased from Proteintech Co., Ltd (Wuhan, China). FIGN antibody was purchased from CUSABIO TECHNOLOGY Co., Ltd (Wuhan, China). Goat anti-rabbit IgG (H+L) (AF488) and Goat anti-mouse IgG (H+L) (AF594) was purchased from ZEN-BIOSCIENCE Co., Ltd. (Chengdu, China).

Oocytes were matured in IVM medium for 24 h at 37 °C, 5% CO_2_, with or without 10^−6^ M NADH. After maturation, oocytes were fixed with 4% PFA for 30 minutes at room temperature, permeabilized with 5% saponin in 5% BSA blocking buffer, and incubated with primary antibodies at specified dilutions (1:50 for mitochondrial subunits; 1:100 for GAS6, FIGN, C14orf39, DMC1; 1:200 for CDK2, FAM9B) overnight at 4 °C. After PBS washing, samples were incubated with species-matched fluorescent secondary antibodies (1:200) for 1 hour under dark conditions. Specimens were mounted in PBS droplets and imaged using a Zeiss LSM 800 confocal microscope with a 20×/0.8 NA objective, maintaining consistent acquisition parameters. Fluorescence intensity quantification was performed using ZEN 3.0 software (Zeiss) with standardized ROIs, with three independent replicates analyzed (including at least three COH cycles).

### 2.11 CDK2 inhibitor and GAS6 protein addition experiments

Immature oocytes obtained through COH cycle were randomly allocated into IVM culture medium with or without CDK2 inhibitor (MedChemExpress, NJ, USA), NADH, and NADH + CDK2 inhibitor. Oocyte maturation progression was quantitatively assessed at 24- and 48-hour time points through morphological evaluation of polar body extrusion. For GAS6 protein (MedChemExpress) addition experiment, immature oocytes collected from COH cycles were randomly divided into IVM medium with or without GAS6 protein. After 24 hours’ culture, ICSI was performed on MII oocytes using standardized clinical protocols, followed by serial embryo developmental assessments with daily morphological scoring. All experiments were conducted in triplicate biological replicates, with temperature-controlled incubation at 37 °C under 5% CO_2_ atmosphere.

### 2.12 Statistical analysis

Categorial variables (maturation/fertilization rates) were analyzed by χ² test with Yates’ correction, while continuous variables (fluorescence intensity) employed Student’s t-test or ANOVA with Bonferroni *post-hoc* analysis. GraphPad Prism 8.0 software generated all graphical outputs, maintaining α = 0.05 significance thresholds.

## 3 Results

### 3.1 Appropriate concentration of NADH promotes oocyte maturation and embryonic development *in vitro*

**Figure 2:**
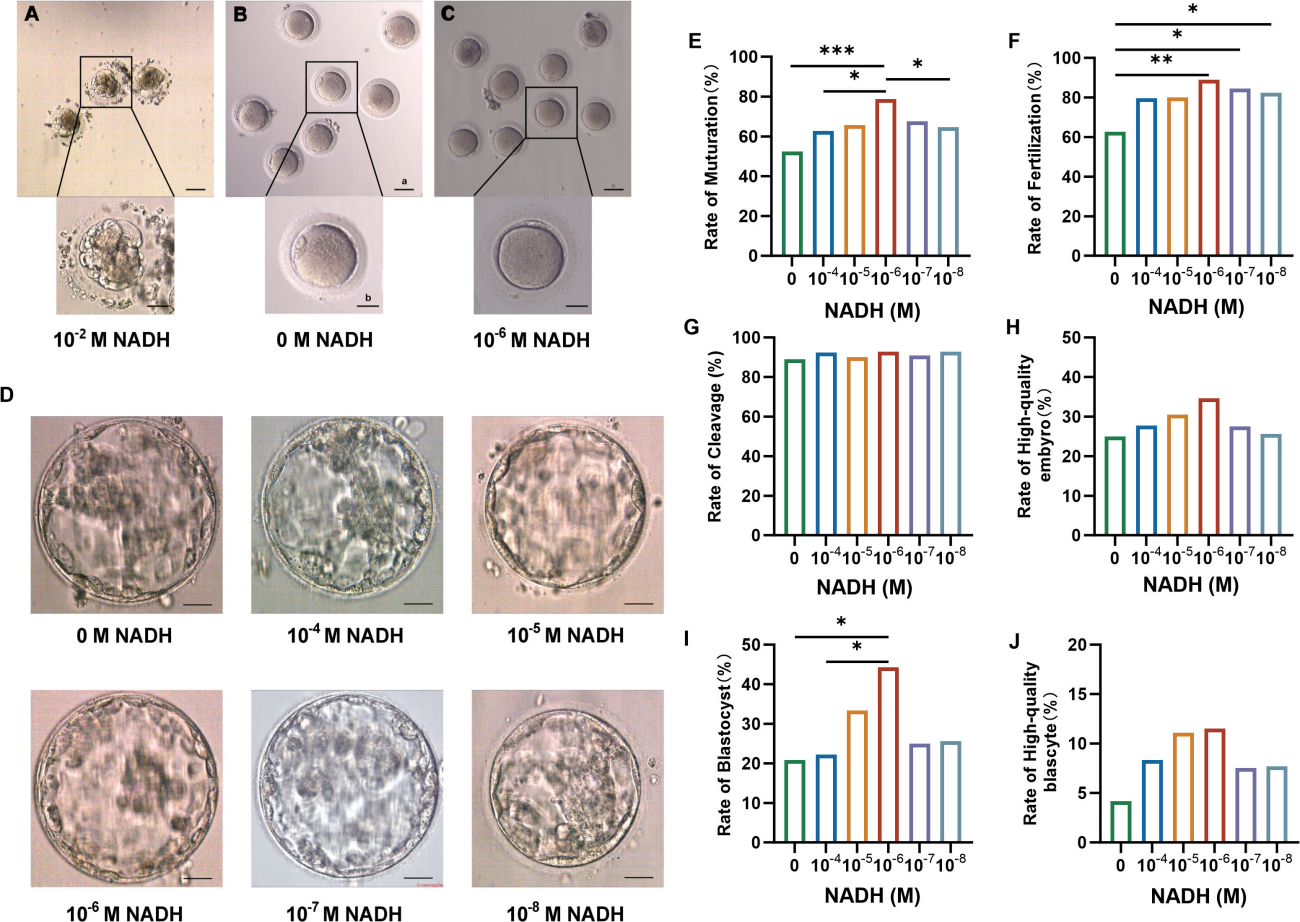
**(A–C)** Oocytes morphology after 24 hours IVM. **(A)** Oocytes cultured with 10^–2^ M NADH added in IVM medium. **(B)** Oocytes cultured without NADH added to IVM medium. **(C)** Oocytes cultured with 10^–6^ M NADH added in IVM medium. Scale bar a, 50 μm; Scale bar b, 25 μm. **(D)** Representative high-quality blastocyst in each group. Scale bar, 50 μm. **(E–J)** Maturation and fertilization of oocytes and embryo development after fertilization in each group. **(E)** The rate of maturation. **(F)** The rate of fertilization. **(G)** The rate of cleavage. **(H)** The rate of high-quality embryo. **(I)** The rate of blastocyst. **(J)** The rate of high-quality blastocyst. *P < 0.05, **P < 0.01, ***P < 0.001.

In preliminary experiments, all oocytes exposed to 10^–2^ M NADH turned black and underwent apoptosis (**Figure 2A**), indicating that high concentrations of NADH negatively impact oocyte development. In the control group without NADH, oocytes cultured for 24 hours exhibited rough cytoplasm, fragmented first polar body, and fragmented perifollicular space (**Figure 2B**). In contrast, most oocytes treated with 10^–6^ M NADH displayed clear cytoplasm, smooth round first polar bodies, and no debris in the perifollicular space, resembling normal mature oocytes *in vivo* (**Figure 2C**).

Subsequently, discarded immature oocytes from COH cycles were randomized into six NADH groups, each with different concentrations for *in vitro* culture and maturation. MII oocytes underwent ICSI and embryo culture. The results are shown in **Table 1**. The 10^–6^ M subgroup exhibited the most favorable indicators, leading to the conclusion that this concentration represents the optimal NADH level. Pairwise comparisons were performed between all NADH concentrations and controls. Statistical analysis revealed that, compared to the control group (0 M NADH), the maturation rate of immature oocyte, fertilization rate, and blastocyst formation rate in the 10^–6^ M group were significantly higher, with statistically significant differences observed (**Figures 2E–J**). Representative images of high-quality blastocysts from each subgroup are presented in **Figure 2D**.

**Table 1:** Effects of different concentrations of NADH on maturation and embryonic development of immature oocytes.

Index	0 M	10^–4^ M	10^–5^ M	10^–6^ M	10^–7^ M	10^–8^ M
No. of immature oocytes (GV)	57	52	50	56	51	52
No. of immature oocytes (MI)	25	26	26	24	26	27
No. of immature oocyte (GV+ MI)	82	78	76	80	77	79
Rate of maturation (%)	52.44% (43/82)	62.82% (49/78)	65.79% (50/76)	78.75% (63/80)	67.53% (52/77)	64.56% (51/79)
Rate of fertilization (%)	62.79% (27/43)	79.59% (39/49)	80.00% (40/50)	88.89% (56/63)	84.62% (44/52)	82.35% (42/51)
Rate of cleavage (%)	88.89% (24/27)	92.31% (36/39)	90.00% (36/40)	92.86% (52/56)	90.91% (40/44)	92.86% (39/42)
Rate of high-quality embryo (%)	25.00% (6/24)	27.78% (10/36)	30.56% (11/36)	34.62% (18/52)	27.50% (11/40)	25.64% (10/39)
Rate of blastocyst (%)	20.83% (5/24)	22.22% (8/36)	33.33% (12/36)	44.23% (23/52)	25.00% (10/40)	25.64% (10/39)
Rate of high-quality blastocyst (%)	4.17% (1/24)	8.33% (3/36)	11.11% (4/36)	11.54% (6/52)	7.50% (3/40)	7.69% (3/39)

Rate of maturation: the number of *in-vitro* matured oocytes (IVM-MII)/the number of immature oocytes (GV + MI). Rate of fertilization: the number of fertilized oocytes/the number of mature oocytes. Rate of cleavage: the number of cleaved embryos/the number of fertilized oocytes. Rate of high-quality embryo: the number of high-quality embryos on day 3/the number of cleaved embryos. Rate of blastocyst: the number of blastocysts/the number of cleavage embryos. Rate of high-quality blastocyst: the number of high-quality blastocysts/the number of cleavage embryos.

**Figure 3:**
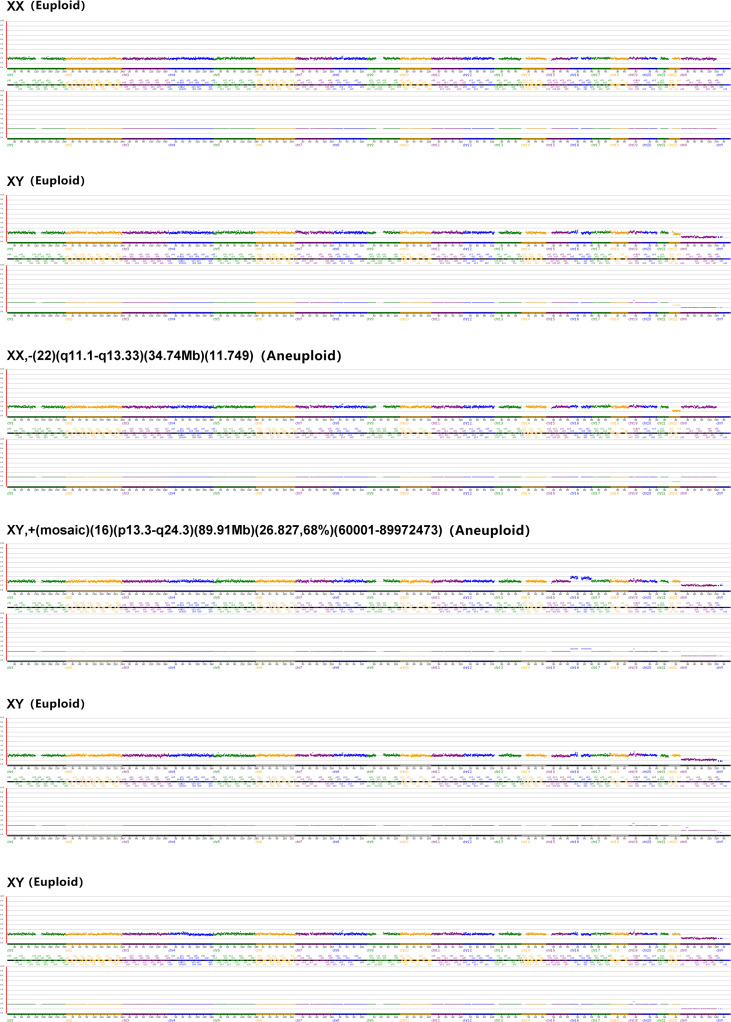
Array CGH results of 6 high-quality blastocysts in the 10^–6^ M NADH group.

To preliminarily assess genetic safety, array comparative genomic hybridization (aCGH) was performed on six high-quality blastocysts derived from the 10^–6^ M NADH group – the subtype typically selected for clinical transfer. Results indicated four euploid and two aneuploid blastocysts (euploid rate: 66.7%, 4/6) (**Figure 3**). This euploidy rate aligns with reported ranges for conventional *in vitro* fertilization (IVF) blastocysts in young patients (52.3%-73.6% euploidy in women <34 years) (28). While encouraging, large cohorts are needed to definitively assess genetic safety. These collective findings establish 10^–6^ M as the optimal NADH concentration for enhancing IVM outcomes without compromising chromosomal integrity, warranting its application in subsequent mechanistic studies.

### 3.2 NADH promotes mitochondrial function in human IVM-MⅡ oocytes

**Figure 4:**
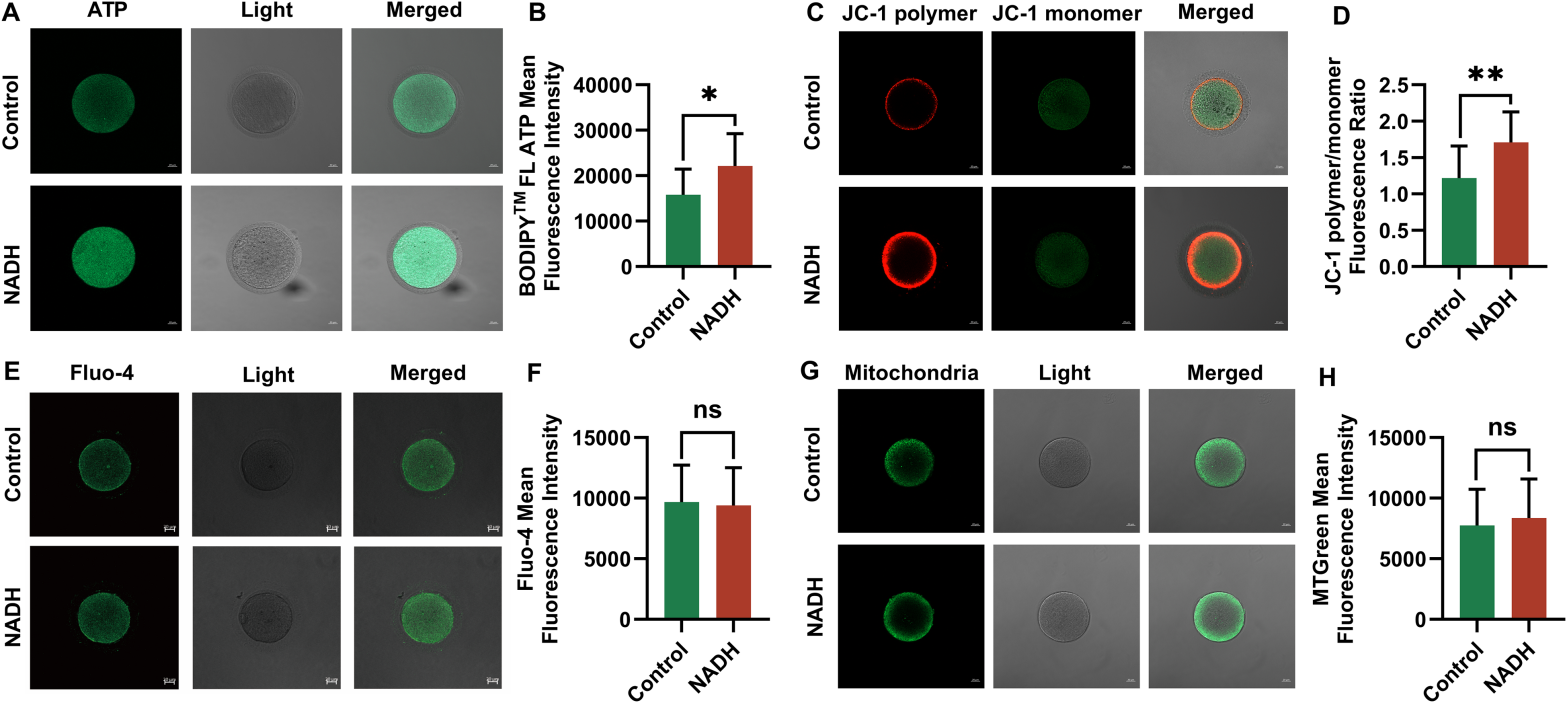
Fluorescence images of human IVM-MⅡ oocytes by laser confocal microscopy and statistical results of repetitive laser confocal assay experiments on the effect of 10^–6^ M NADH. Scale bar, 20 μm. **(A, B)** Effect of NADH on ATP in human IVM-MⅡ oocytes. Sample size: Control group: n = 13; NADH group: n = 13. **(C, D)** Effect of NADH on MMP in human IVM-MⅡ oocytes. Sample size: Control group: n = 15; NADH group: n = 15. **(E, F)** Effect of NADH on Ca^2+^ in human IVM-MⅡ oocytes. Sample size: Control group: n = 17; NADH group: n = 17. **(G, H)** Effect of NADH on the mitochondrial content in human IVM-MⅡ oocytes. Sample size: Control group: n = 20; NADH group: n = 22. *P < 0.05, **P < 0.01.

The mitochondrial function of the oocytes will be disturbed during *in vitro* culture and maturation. The main indicators of mitochondrial function are MMP, Ca^2+^ level and ATP content. Our study demonstrated that the ATP levels (P < 0.05, **Figures 4A, B**) and levels of MMP (P < 0.01, **Figures 4C, D**) in IVM-MII (metaphase II) oocytes, cultured for 24 hours following the addition of 10^–6^ M NADH, were significantly elevated compared to those in oocytes that did not receive NADH supplementation. The Ca^2+^ levels of IVM-MII oocytes cultured with NADH were slightly lower compared to those of oocytes without NADH addition (**Figures 4E, F**). Additionally, the mitochondrial content in oocytes with NADH was slightly higher than in those without NADH addition (**Figures 4G, H**). However, these differences were not statistically significant. These results indicated that 10^–6^ M NADH enhanced the developmental potential of IVM-MII oocytes by improving mitochondrial function and increasing ATP production, although it did not increase mitochondrial quantity.

In addition, oocytes generate ROS as a result of exposure to visible light, ambient oxygen, and endogenous metabolic activity. Excessive ROS can disturb the oxidative balance within cells, impair mitochondrial function, decrease fertilization rates, and negatively impact embryonic development (29). GSH functions as an antioxidant, mitigating oxidative stress by eliminating excess ROS. Reduced levels of GSH are indicative of the early activation of apoptosis (30). To investigate whether 10^–6^ M NADH can maintain the balance of ROS and GSH in oocytes, we measured ROS and GSH levels in IVM-MII oocytes with and without NADH supplementation using confocal laser microscopy.

**Figure 5:**
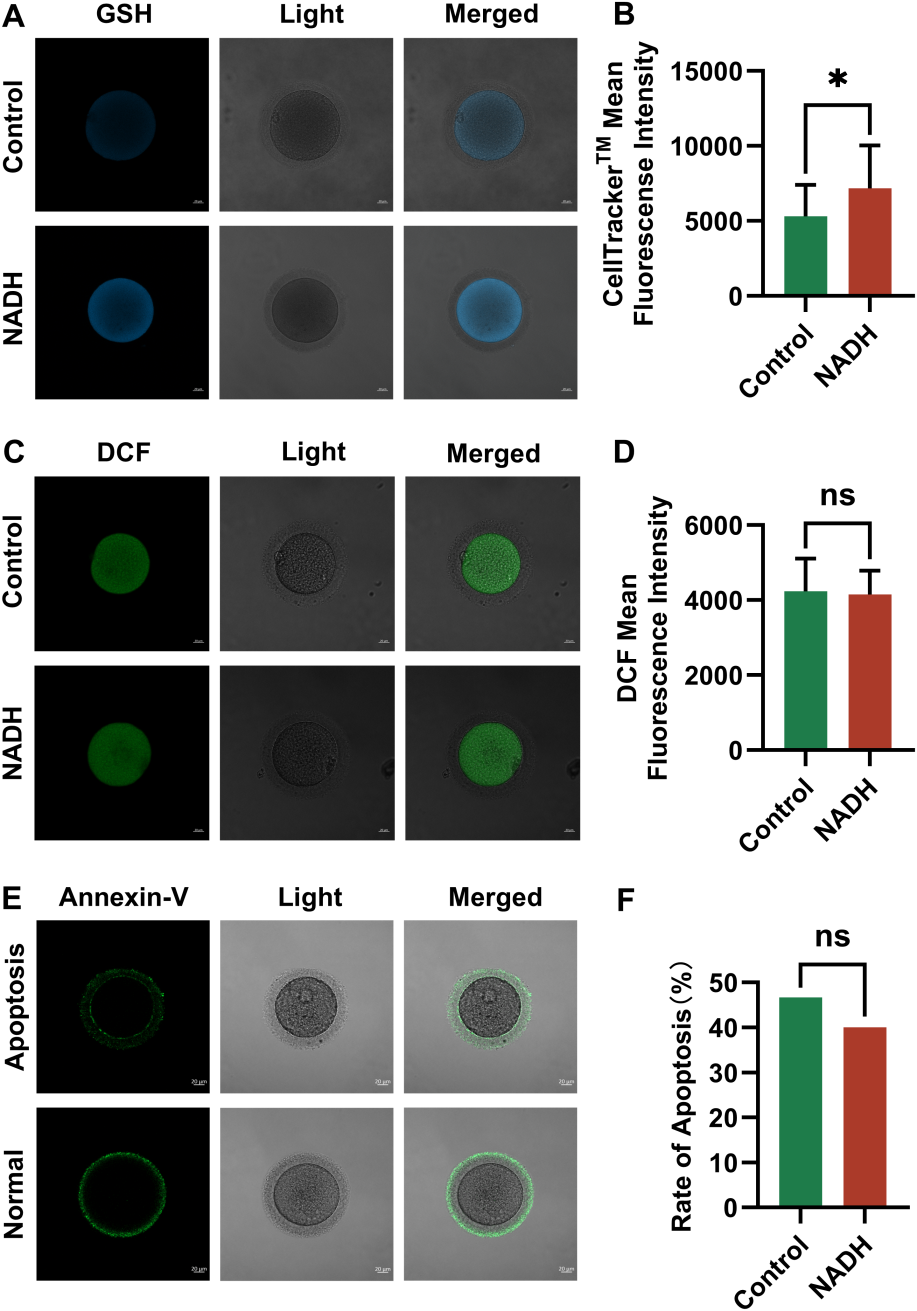
Fluorescence images of human IVM-MⅡ oocytes by laser confocal microscopy and statistical results of repetitive laser confocal assay experiments on the effect of 10^–6^ M NADH. Scale bar, 20 μm. **(A, B)** Effect of NADH on GSH in human IVM-MⅡ oocytes. Sample size: Control group: n = 19; NADH group: n = 18. **(C, D)** Effect of NADH on ROS in human IVM-MⅡ oocytes. Sample size: Control group: n = 30; NADH group: n = 28. **(E, F)** Effect of NADH on early apoptosis of human IVM-MⅡ oocytes. The Annexin-V green fluorescent signal was displayed on both the zona pellucida and the oocyte membrane, which represented early apoptosis of the oocyte, and only the zona pellucida showed the green fluorescence signal of Annexin-V, and the cell membrane did not show the green fluorescence signal, which represented the normal oocyte. Sample size: Control group: n = 30, early apoptosis: n = 14; NADH group: n = 30, early apoptosis: n = 12. *P < 0.05.

Multiple experiments demonstrated GSH levels were significantly increased (P < 0.05) in the NADH group (**Figures 5A, B**) However, there were no significant difference in ROS levels between the 10^–6^ M NADH-treated oocytes cultured for 24 hours and the control group without NADH (**Figures 5C, D**). Given the established relationship between oxidative stress and oocyte apoptosis, this study also evaluated the apoptosis rates of mature oocytes in both groups, revealing no significant difference in apoptosis rates (**Figures 5E, F**). These findings suggested that the increased GSH levels in the NADH group effectively reduce ROS levels in oocytes, thereby alleviating oxidative stress.

What’s more, given the role of electron transport chain complex I (ETC CI) in ATP synthesis and NADH as its primary substrate, we hypothesized that supplementing IVM media with NADH might enhance the activity of ETC complex I during oocyte maturation. To explore potential changes in CI, immature oocytes underwent 24-hour IVM in media supplemented with or without 10^–6^ M NADH, followed by immunofluorescence (IF) staining for key CI subunits (NDUFV1, NDUFS7, DAP13, ND1). We acknowledge IF provides spatial/semi-quantitative data; absolute quantification would require Western blotting (WB), which was precluded by limited oocyte availability from COH cycles. The 24-hour timeframe was selected to allow for potential protein-level adaptations (e.g., synthesis, degradation, or complex assembly). However, no significant differences in IF signal intensity or localization of these subunits were observed between NADH-treated and control groups (Appendix A: **Supplementary Figure S1**). Critically, unchanged CI subunit abundance does not preclude altered enzymatic activity (e.g., via post-translational modifications). Thus, while NADH supplementation during IVM did not affect detectable CI protein levels, its impact on CI activity warrants direct assessment in future.

### 3.3 Impact of NADH on the expression profile of human IVM- MⅡ oocyte transcriptome

**Figure 6:**
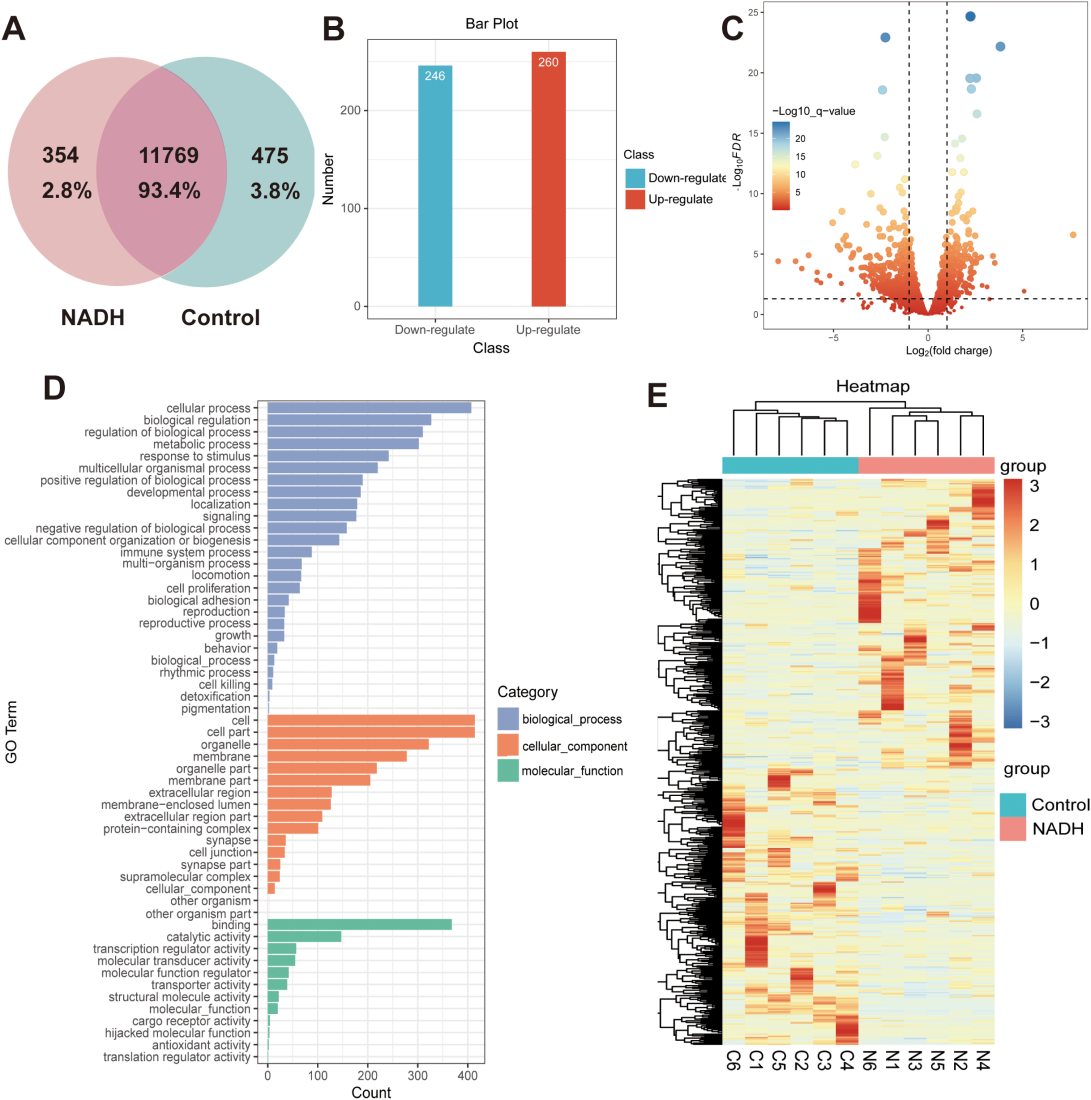
**(A–E)** Whole transcriptome analysis in single oocyte from Control and NADH group with 10^–6^ M. **(A)** Vene analysis. **(B)** Bar plot showing the number of DEGs. Red: up-regulated; blue: down-regulated. **(C)** Volcano plot showing DEGs in NADH oocytes compared with Control oocytes. **(D)** GO enrichment analysis on DEGs. Blue bars refer to the terms related to biological process; orange bars refer to the terms related to cellular component; and green bars refer to the terms related to molecular function. **(E)** Heatmap of the DEGs related to the oocyte maturation.

To further investigate the mechanism by which NADH influences the development of human immature oocytes, this study employed single-cell transcriptome sequencing technology to analyze the gene expression profile. Vene analysis identified differentially expressed genes (DEGs) between the NADH group and the control group (**Figure 6A**). Compared with the control group, approximately 506 genes were abnormally expressed in the NADH group, including 260 up-regulated genes and 246 down-regulated genes, among which DEGs were determined by screening criteria [fold charge ≥ 2.0 (or-2.0), P < 0.05] (**Figures 6B, C**). Gene Ontology (GO) enrichment analysis showed that “cellular process” was the most significant in “Biological Process”. Within the “Cellular Component” category, “cell” was the most important, while within the “Molecular Function” category, “binding” was the most prominent (**Figure 6D**). Next, genes related to maturation, metabolism and immune were listed in the heat map (**Figure 6E**). Among the DEGs, six up-regulated genes related to oocyte maturation were screened and identified, from high to low: FIGN, FAM9B, GAS6, C14orf39, DMC1 and CDK2 (Appendix B.2: **Supplementary Table S2**).

**Figure 7:**
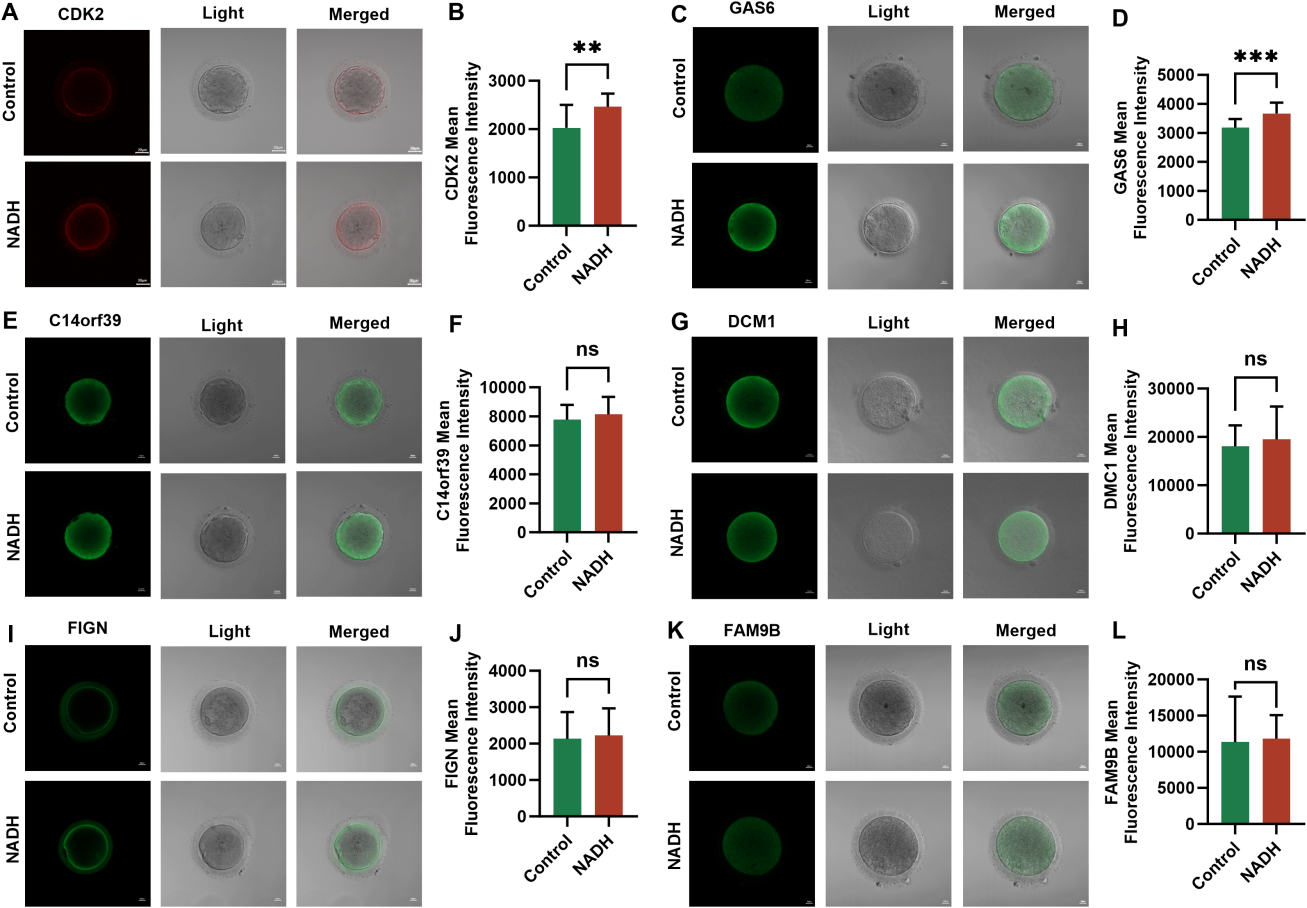
Fluorescence images of human IVM-MⅡ oocytes by laser confocal microscopy and statistical results of repetitive laser confocal assay experiments on the effect of 10^–6^ M NADH. Scale bar, 20 μm. **(A, B)** Effect of NADH on CDK2 gene in IVM-MⅡ oocytes. Sample size: Control group: n = 17; NADH group: n = 18. **(C, D)** Effect of NADH on GAS6 gene in IVM-MⅡ oocytes. Sample size: Control group: n = 17; NADH group: n = 15. **(E, F)** Effect of NADH on C14orf36 gene in IVM-MⅡ oocytes. Sample size: Control group: n = 14; NADH group: n = 16. **(G, H)** Effect of NADH on DCM1 gene in IVM-MⅡ oocytes. Sample size: Control group: n = 15; NADH group: n = 18. **(I, J)** Effect of NADH on FIGN gene in IVM-MⅡ oocytes. Sample size: Control group: n = 15; NADH group: n = 24. **(K, L)** Effect of NADH on FAM9B gene in IVM-MⅡ oocytes. Sample size: Control group: n = 17; NADH group: n = 17. **P < 0.01, ***P < 0.001.

The expression differences of these six genes between the NADH group and the control group were further verified by immunofluorescence experiments. The results indicated that the expressions of CDK2 (P < 0.01) (**Figures 7A, B**) and GAS6 (P < 0.001) (**Figures 7C, D**) genes in the NADH group were significantly higher than those in the control group, demonstrating statistical significance. Although the expression of the other four genes was elevated in the NADH group, the difference compared to the control group were not statistically significant (**Figures 7E–L**). The results showed that NADH was likely to play an important role in the oocyte meiosis process and cytoplasmic maturation, thereby improving the quality of IVM oocytes.

### 3.4 NADH enhances human oocyte maturation *in vitro* by upregulating the CDK2 and GAS6 genes

**Figure 8:**
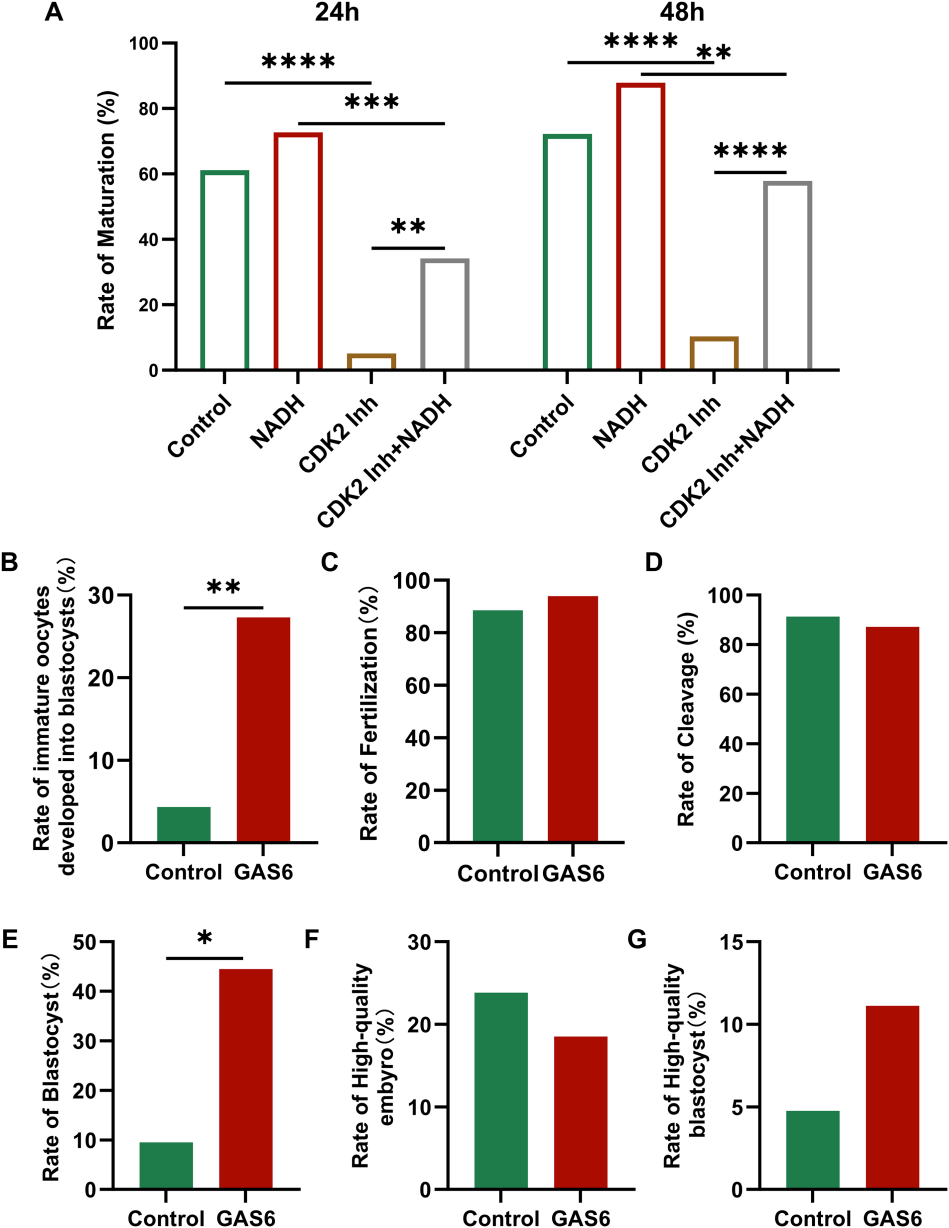
**(A)** Maturation rate of immature oocytes collected in COH under different culture conditions. Inh: Inhibitor. **(B–G)** Fertilization of oocytes and embryonic development after fertilization in each group. **(B)** Rate of immature oocytes developed into blastocysts. **(C)** The rate of fertilization. **(D)** The rate of cleavage. **(E)** The rate of blastocyst. **(F)** The rate of high-quality embryo. **(G)** The rate of high-quality blastocyst. *P < 0.05, **P < 0.01, ***P < 0.001, ****P < 0.0001. Inh: inhibitor.

CDK2 plays a crucial role in regulating cell cycle progression, particularly during the transition from the G1 to S phase. To further investigate its role, we designed the following experiment: immature oocytes were collected from the COH cycle and randomly assigned to different treatment conditions. The maturation rates of the cells were then observed after 24 and 48 hours (Appendix B.3: **Supplementary Table S3**). In the 24-hour culture (**Figure 8A**, left), the maturation rate of the group treated with the CDK2 inhibitor was significantly reduced (P < 0.0001). In contrast, the maturation rate of the group receiving both the CDK2 inhibitor and NADH was improved compared to the CDK2 inhibitor alone (P < 0.01). After 48 hours of culture (**Figure 8A**, right), the maturation rate in the CDK2 inhibitor and NADH group increased even more significantly (P < 0.0001). Although CDK2 inhibition is known to disrupt meiosis, NADH supplementation significantly restored maturation rates (24h: P<0.01; 48h: P<0.0001 vs. inhibitor-only group; **Figure 8A**). This implies NADH may overcome CDK2 blockade by boosting compensatory pathways (e.g., CDK1 activation or metabolic support).

GAS6 is a natural ligand for the TAM receptor tyrosine kinase (Tyro3/Axl/MerTK), and the γ-carboxyglutamate (Gla) domain in its structure is targeted to the cell membrane by binding to phosphatidylserine, activating the downstream PI3K/Akt and MAPK signaling pathways in a paracrine/autocrine form (31). To verify their function, immature oocytes were randomly divided into control group and exogenous recombinant human GAS6 protein treatment group (32). After 24 hours’ culture, mature oocyte was performed by ICSI to assess embryonic developmental outcomes (Appendix B.4: **Supplementary Table S4**). The results showed that the rate of immature oocytes developed into blastocysts in the GAS6-treated group was significantly higher (**Figure 8B**, P < 0.01). However, the fertilization rate (**Figure 8C**) and cleavage rate (**Figure 8D**) showed no significant changes. Additionally, the rate of blastocyst in the GAS6 treated group was significantly increased (**Figure 8E**, P < 0.05), while there were no significant differences observed in the proportions of high-quality embryos (**Figure 8F**) and high-quality blastocysts (**Figure 8G**). These findings suggested that GAS6 may facilitate late embryonic development.

## 4 Discussion

Oocyte maturation represents a coordinated interplay between nuclear and cytoplasmic events, with mitochondrial bioenergetics serving as the cornerstone of developmental competence (33). While IVM offers a promising strategy for infertility treatment, its clinical application remains limited by delayed cytoplasmic maturation—particularly in aging populations where mitochondrial dysfunction drives poor outcomes (34). As a crucial molecule in energy metabolism, NADH is intricately linked to mitochondrial function. Consequently, NADH holds promise for applications aimed at enhancing mitochondrial function and improving oocyte developmental capacity.

Under physiological conditions, the generation of ROS in oocytes, together with the Ca^2+^ level in the endoplasmic reticulum (ER), the generation of mitochondrial ATP and MMP, forms a cyclic closed loop to maintain the stability of oxidative stress levels in oocytes (35). Mechanistically, NADH elevated ATP production and MMP without increasing ROS, a paradoxical phenomenon explained by its dual role as an electron donor and redox buffer. This contrasts with conventional NAD^+^ precursors (e.g., NMN/NR) that primarily target NAD^+^ salvage pathways (9). The observed GSH upregulation (P < 0.05) likely stems from NADH-driven NADP^+^ conversion via NAD kinase, fueling glutathione synthesis (36), while its direct antioxidant capacity neutralizes ROS generated during accelerated ATP synthesis (37). The most direct way to demonstrate NADH antioxidant pathway activation is to measure the NADPH/NADP^+^ ratio in oocytes. However, due to the scarcity of samples, it is difficult to complete the indicators of human oocytes, and we can only indirectly speculate through the existing indicators. In the future, we will continue to improve experiments on animal models that are difficult to achieve with human oocytes. Our IF data showed no alteration in ETC complex I subunit abundance after 24-hour NADH supplementation. However, mitochondrial enzyme activity can be modulated independently of protein levels, such as via phosphorylation, acetylation, or altered substrate availability (38, 39). Thus, NADH could still enhance ETC complex I activity during IVM – a hypothesis requiring validation via functional assays.

At the transcriptional level, single-cell RNA sequencing unveiled NADH’s unique capacity to independently upregulate CDK2 and GAS6—two master regulators of oocyte competence. CDK2 encodes a serine/threonine protein kinase that is crucial for cell cycle progression, particularly for the transition from the G1 to S phase. Inhibition of CDK2 can activate the spindle assembly checkpoint, impede cell cycle progression, and disrupt the completion of oocyte meiosis (40). Bertondo et al. unequivocally demonstrated that NAD^+^ supplementation reverses aging-associated fertility decline by activating sirtuins, especially SIRT2, through mechanisms including restoring mitochondrial function and reducing DNA damage. This study pointed out that NAD^+^ is an essential cofactor for sirtuins, and that sirtuins deacetylation regulates downstream gene expression (25). Pollard et al. pointed out that NAD^+^ supplementation increased granulosa cell proliferation and oocyte maturation, and CDK2 was the core factor driving G1/S phase transition. NAD^+^ regulates cyclins (e.g., Cyclin E) through sirtuins, which may be a pathway for CDK2 activation (41). Liang et al. clarified that NAD^+^ precursors maintain the integrity of the meiotic spindle through sirtuins, and CDK2 is involved in the regulation of meiosis, suggesting a functional relationship between the two (42). They also emphasize that NR activates sirtuins by maintaining NAD^+^ levels, thereby protecting oocyte spindle structure and chromosomal stability, and indirectly affects cell cycle regulators such as CDK2. Goruppi et al. showed that GAS6, acting as an Axl receptor ligand, promotes cell cycle entry (e.g., S phase) and inhibits apoptosis by activating the MAPK pathway (43). Smits et al. noted that NAD^+^ deficiency impairs energy metabolism, and AMPK, as a cellular energy sensor, often synergistically regulates metabolism with NAD^+^/sirtuins (e.g., SIRT1-AMPK interaction) (44). Therefore, sirtuins deacetylated transcription factors (e.g., FOXO, p53) may directly promote GAS6 transcription. It can be seen that NADH may be dependent on the sirtuins pathway (especially SIRT2) to upregulate CDK2 and GAS6 through the conversion to NAD^+^, and this inference needs to be further verified. Besides, the selective improvement in blastocyst rates—uncoupled from Day 3 embryo quality—underscores GAS6’s role in post-compaction events. During blastulation, GAS6/TAM receptor signaling mitigates oxidative stress-induced apoptosis and promotes trophoblast differentiation (45). This stage-specific action mirrors the metabolic shift from glycolysis to oxidative phosphorylation required for cavitation (46). Future studies will delineate whether GAS6 optimizes mitochondrial function or epigenetic reprogramming during this critical transition.

In addition to the CDK2 and GAS6 genes, the results of single egg sequencing also found that these four genes (C14orf39, DMC1, FIGN, FAM9B) were also associated with maturation and were up-regulated. Although the immunofluorescence results of these genes did not differ between the NADH and control groups, their potential role should not be overlooked. C14orf39 is vital for homologous chromosome pairing, DNA double-strand breaks, recombination, and ovulation. The mutation or loss of C14orf39 can result in defects in chromosome synapsis and meiotic arrest, ultimately leading to infertility (47–49). DMC1, a member of the recombinase superfamily, is responsible for repairing DNA double-strand breaks during both mitosis and meiosis; mutations in this gene cause defects in the synapsis complex and meiotic arrest during phase I (50, 51). In mammals, DMC1 plays a crucial role in homologous chromosome recombination and DNA mismatch repair. Its loss negatively affects oocyte maturation and triggers apoptosis (52). FIGN influences meiotic spindle assembly (53), and Li et al. demonstrated that it is abundant in the zona pellucida of mouse oocytes, where it may help prevent polyspermy (54). FAM9B located on the X chromosome is present in the follicular nuclei and granulosa cytoplasm of human ovarian tissue and plays a crucial role in oocyte maturation (55).

Comparing aneuploidy rates in NADH-treated blastocysts (n=6) to historical IVF controls, differences in maternal age, stimulation regimen, or year of collection may confuse interpretation. This preliminary analysis requires further validation of the safety of NADH for clinical use through prospective randomized trials. For ongoing studies, we have implemented cryopreservation of control embryos from consenting patients to enable matched contemporaneous analysis. In addition, our data derive from COH-cycle oocytes, the current standard for clinical IVM. Caution is warranted in extrapolating these results to natural cycles or pathological contexts (e.g., PCOS, advanced age), where distinct metabolic states may alter NADH efficacy. Validating our findings in these populations is essential for broader application.

MT remains a robust antioxidant for IVM (56), NADH offers unique advantages for oocytes with bioenergetic deficits. Combining NADH with melatonin or other antioxidants (e.g., resveratrol) represents a promising IVM optimization strategy. The complementary profiles of these agents suggest therapeutic synergy: MT could prevent oxidative damage during mitochondrial expansion, while NADH maximizes ATP yield from newly formed organelles. Future studies should titrate combination ratios to balance efficacy and safety. We are currently designing such trials using murine models to identify synergistic pairs before human testing. There are some limitations inherent in our NADH’s research. Sample sizes for some assays were constrained by the limited availability of discarded immature oocytes per patient. These factors necessitate cautious interpretation of negative results. Future large-scale collaborations are essential to confirm these findings. The validation of CDK2 and GAS6 proteins is not based on a direct mechanism to verify causation, which is a limitation in our study. Future studies need to detect CDK2 kinase activity in NADH-treated oocytes and disrupt GAS6 signaling with AXL receptor blockers. These approaches are underway in our lab using murine models permitting larger-scale sampling.

## 5 Conclusions

**Figure 9:**
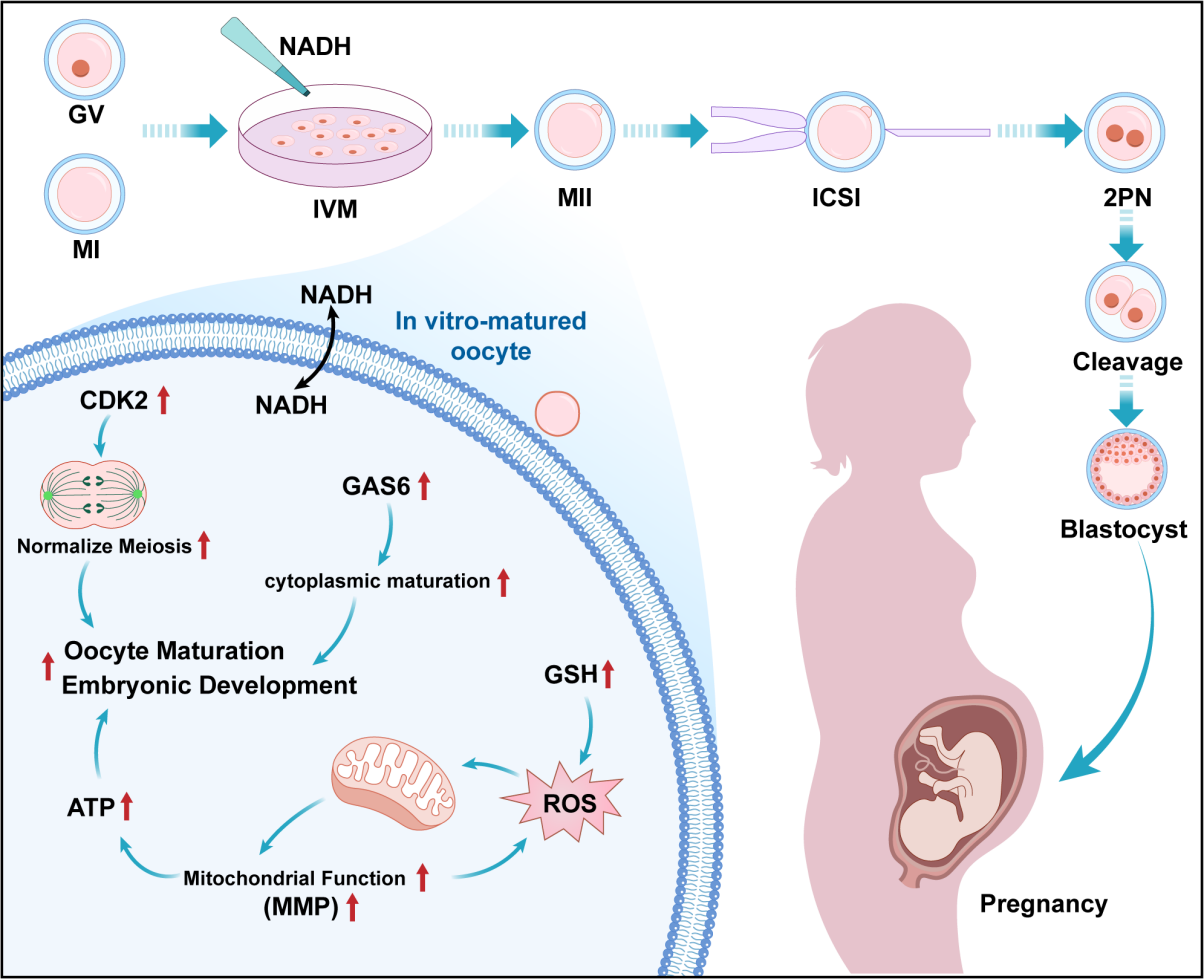
Working model for the impact of NADH on the quality of COH immature oocytes. Presented new findings on IVM culture of human immature oocytes during the COH cycle: The addition of 10^–6^ M NADH in IVM medium can effectively improve the developmental potential of IVM-MⅡ oocytes. The mechanism is that the application of NADH significantly increases the levels of ATP, MMP and GSH in oocytes, and realizes the improvement of mitochondrial function and antioxidant capacity of IVM- MⅡ oocytes. NADH up-regulates CDK2 and GAS6 genes, protected the cell cycle progression and promoted cytoplasmic maturation and facilitates embryonic development respectively.

This pilot study provides initial evidence that NADH supplementation improves human oocyte maturation in COH cycles (see **Figure 9**). Key observations include: NADH (10^−6^ M) elevates mitochondrial bioenergetics and developmental outcomes; Transcriptional profiling associates NADH with upregulation of CDK2 (cell cycle) and GAS6 (cytoplasmic maturation); Intervention experiments show CDK2 inhibition impairs maturation, partially alleviated by NADH and exogenous GAS6 enhances blastocyst formation. While these findings implicate CDK2/GAS6 as potential mediators, functional validation is needed to establish causality. Future studies should validate CDK2/GAS6 functional necessity (e.g., siRNA in cumulus-oocyte complexes); define NADH’s upstream signaling (e.g., SIRT1-AMPK axis); Assess clinical translatability in larger cohorts.

Collectively, NADH emerges as a candidate IVM enhancer whose bioenergetic-transcriptional interplay warrants deeper exploration.

## Data Availability

The original contributions presented in the study are included in the article/**Supplementary Material**. Further inquiries can be directed to the corresponding authors.

## Supplementary material

Supplementary Figure 1 Fluorescence images of human IVM-MⅡ oocytes by laser confocal microscopy and statistical results of repetitive laser confocal assay experiments on the effect of 10^–6^ M NADH. Scale bar, 20 μm **(A)** Effect of NADH on NDUFV1 protein expression in human IVM-MⅡ oocytes. Sample size: Control group: n = 14; NADH group: n = 13. **(B)** Effect of NADH on DAP13 protein expression in human IVM- MⅡ oocytes. Sample size: Control group: n = 12; NADH group: n = 13. **(C)** Effect of NADH on NDUFS7 protein expression in human IVM- MⅡ oocytes. Sample size: Control group: n = 10; NADH group: n = 11. **(D)** Effect of NADH on DAP13 protein expression in human IVM- MⅡ oocytes. Sample size: Control group: n = 10; NADH group: n = 10.

Supplementary Table 1 Baseline level of female patients in each group. BMI, body mass index; FSH, follicle-stimulating hormone; E2, estrogenic hormone; P, pregestational hormone; PRL, prolactin; LH, luteinizing hormone; T, testosterone. All data are expressed as mean ± S.

Supplementary Table 2 Differentially expressed genes associated with oocyte maturation.

Supplementary Table 3 Effects of different concentrations of CDK2 inhibitor on oocyte maturation. Rate of maturation: the number of *in-vitro* matured oocytes (IVM-MⅡ)/the number of immature oocytes (GV+MІ).

Supplementary Table 4 Embryonic development in the GAS6 protein supplemented group and the non-supplemented group. Rate of immature oocyte development into blastocyst (%): the number of blastocysts/the number of immature oocytes. Rate of fertilization: the number of fertilized oocytes/the number of mature oocytes. Rate of cleavage: the number of cleaved embryos/the number of fertilized oocytes. Rate of high-quality embryo: the number of high-quality embryos on day 3/the number of cleaved embryos. Rate of blastocyst: the number of blastocysts/the number of cleavage embryos. Rate of high-quality blastocyst: the number of high-quality blastocysts/the number of cleavage embryos.
