# Supplementary material for: NADH supplementation improves human oocyte maturation and developmental competence of resulting embryos in controlled ovarian hyperstimulation cycles: a pilot study implicating the CDK2/GAS6 signaling pathway
Source: Front Endocrinol (Lausanne). 2025 Sep 3;16:1627679. doi: 10.3389/fendo.2025.1627679 (PMC12440754; doi:10.3389/fendo.2025.1627679)
Supplement: Supplementary Figure 1 — Fluorescence images of human IVM-MⅡ oocytes by laser confocal microscopy and statistical results of repetitive laser confocal assay experiments on the effect of 10–6 M NADH. Scale bar, 20 μm (A) Effect of NADH on NDUFV1 protein expression in human IVM-MⅡ oocytes. Sample size: Control group: n = 14; NADH group: n = 13. (B) Effect of NADH on DAP13 protein expression in human IVM- MⅡ oocytes. Sample size: Control group: n = 12; NADH group: n = 13. (C) Effect of NADH on NDUFS7 protein expression in human IVM- MⅡ oocytes. Sample size: Control group: n = 10; NADH group: n = 11. (D) Effect of NADH on DAP13 protein expression in human IVM- MⅡ oocytes. Sample size: Control group: n = 10; NADH group: n = 10. [file DataSheet1.docx]

**IVM medium preparation**

The base medium consisted of Tissue Culture Medium 199 (Gibco™, Cat# 11150059) supplemented with:

- 0.22 mM sodium pyruvate (Sigma-Aldrich, Cat# P2256)
- 0.075 IU/mL recombinant human FSH (Gonal-f®)
- 0.5 IU/mL urinary hCG (Livzon®)
- 10 μg/mL 17β-estradiol (E2)
- 0.6 g/L penicillin-streptomycin antibiotic cocktail (Gibco™, Cat# 15140122)
- 20% (v/v) autologous serum (collected on COH day 2, processed via 200 × g centrifugation for 10 min, heat-inactivated at 56°C for 30 min, and sterilized through 0.22 μm filtration)

**IVM, ICSI, and embryo culture**

Oocyte Preparation:

- Washed twice in Gamete Buffer™ (Cook Medical)
- Cultured in 50 μL IVM microdroplets (5 oocytes/droplet) under mineral oil (Vitrolife, Sweden, Cat# 10029)
- Incubation conditions: 37°C, 6% CO₂, 5% O₂ (humidified tri-gas incubator, ASTEC®)

Maturation Assessment:

- Evaluated at 24 h post-IVM using IX-71 inverted microscope (Olympus Corporation, Tokyo, Japan)
- Maturation confirmation: extrusion of first polar body (PB1)

ICSI Procedure:

- Sperm samples: Normozoospermic partners (WHO 2021 criteria)
- Injection performed using PiezoDrill® system (PrimeTech, Japan)

Embryo Development Monitoring:

- Fertilization check: 16-18 h post-ICSI (2 pronuclei + 2 polar bodies)
- Culture protocol:

Days 1-3: Cleavage Medium™ (20 μL microdroplets)

Days 3-6: Blastocyst Medium™ with daily medium renewal

- Time-lapse documentation: EmbryoScope® system (15-min imaging intervals)

Blastocyst Evaluation:

- Day 5/6 grading: Gardner-Schoolcraft classification system [1]
- Morphokinetic analysis: EmbryoViewer® software (v2.5)

**Blastocyst grading**

1. Developmental Stage Classification

| Stage | Morphological Characteristics |
| --- | --- |
| 1 | Early blastocyst (Blastocoel < 50% embryo volume) |
| 2 | Blastocyst (Blastocoel > 50% volume) |
| 3 | Full blastocyst (Complete blastocoel expansion) |
| 4 | Expanded blastocyst (Zona thinning < 10 μm) |
| 5 | Hatching (TE protrusion through zona) |
| 6 | Hatched (Complete TE-ICM egression) |

1. Trophectoderm (TE) Grading

A: Continuous monolayer of ≥ 30 cohesive cells

B: 15-29 cells with minor discontinuities

C: < 15 cells with significant gaps

1. Inner Cell Mass (ICM) Grading

A: Compact cluster of ≥ 20 tightly adherent cells

B: 10-19 loosely arranged cells

C: < 10 cells with degenerative features

1. High-Quality Blastocyst Definition

Day 5: ≥ 3BB (Expansion stage 3 with both TE and ICM grade B or higher)

Day 6: ≥ 4BB (Expansion stage 4 with both TE and ICM grade B or higher)

**Blastocyst Cryopreservation and Warming Protocol**

**Vitrification Procedure**

The vitrification process was conducted using a two-step exposure protocol. Initially, blastocysts were equilibrated in a solution containing 7.5% (v/v) ethylene glycol and 7.5% (v/v) dimethyl sulfoxide (DMSO) at room temperature for 15 minutes. Subsequently, the embryos were transferred to a vitrification solution comprising 15% ethylene glycol, 15% DMSO, and 0.5 mol/L sucrose for 60 seconds. Using a precision loading technique, 2-3 blastocysts were positioned on the Cryotop carrier (Kitazato Biopharma) within a minimal fluid volume of < 0.1 µL. The loaded Cryotop was immediately plunged into liquid nitrogen (-196 °C) and subsequently sealed with its protective sheath prior to long-term storage in liquid nitrogen tanks.

**Warming and Embryo Recovery**

For blastocyst resuscitation, the Cryotop device was rapidly immersed in pre-warmed (37 °C) thawing solution containing 1 M sucrose. Following initial recovery, the embryos were sequentially transferred through: 1) a 3-minute incubation in 0.5 mol/L sucrose dilution solution, and 2) a 3-minute wash in sucrose-free solution. Surviving blastocysts were subsequently cultured in a triple-gas incubator (37 °C, 6% CO2, 5% O2) with humidified atmosphere until further processing.

Prior to genetic analysis, zona pellucida modification was performed using a non-contact laser system (Hamilton Thorne Laser Systems). A standardized aperture (30-40 µm diameter) was created in the zona pellucida to facilitate extraction of the trophectoderm-inner cell mass (TE-ICM) complex. This microsurgical procedure employed two custom-fabricated elongated glass micropipettes under stereomicroscopic guidance. The isolated TE-ICM complex was subsequently subjected to array-based comparative genomic hybridization (array CGH) for comprehensive aneuploidy screening.

**Array CGH Procotol**

The TE-ICM of each blastocyst was transferred to PBS, washed 2 or 3 times, placed in a microcentrifuge tube containing 2.5 μL PBS, and centrifuged for a few seconds. Amplification of the genomic DNA and whole genome amplification (WGA) were performed as previously described [2, 3]. In brief, the WGA products were fluorescently labeled and competitively hybridized to 24sure V3 arrays (BlueGnome, Cambridge, UK) using a matched control in an array CGH experimental format. A laser scanner (InnoScanw 710 AL, INNOPSYS, Carbonne, France) was used to excite the hybridized fluorophores and read and store the resulting hybridization images. The scanned images were analyzed and quantified by an algorithm at fixed settings using the BlueFuse Multi software (BlueGnome, Cambridge, UK), which is a software package that automatically performs the grid placement, quantification, normalization, and postprocessing steps. The entire procedure was completed within 12-24 hours.

**Single-cell RNA sequencing (scRNA-seq) analysis**

Oocytes from each experimental group (n=6 per group) were subjected to scRNA-seq analysis with three biological replicates. All specimens were cryopreserved at -80 °C in RNA stabilization solution (BGI Shenzhen, China) prior to processing. Total RNA was isolated from IVM-treated oocytes (with or without NADH supplementation) using the RNeasy Plus Mini Kit (Qiagen, Germany) following manufacturer specifications. cDNA library preparation and subsequent sequencing were performed on the BGISEQ-500 platform (BGI Shenzhen).

Read alignment was conducted using a dual-strategy approach: Bowtie2 v2.3.4.3 for reference genome mapping (GCF_000001635.27_GRCm39) and HISAT2 v2.2.1 for transcriptome alignment. Sequencing depth and mapping efficiency were systematically evaluated through read distribution analysis across genomic features. Transcript quantification employed RSEM v1.3.3 with FPKM normalization. For differential expression analysis, we established a minimum expression threshold of FPKM ≥10 across sample groups.

Differentially expressed genes (DEGs) were identified using the NOISeq package with stringent criteria (|fold change| > 2, adjusted P < 0.05). Functional annotation of DEGs was performed through:

1. Gene Ontology (GO) enrichment analysis (GO database release 2023.05)
2. Pathway mapping using the Kyoto Encyclopedia of Genes and Genomes (KEGG release 107.0)
3. Statistical significance for enrichment analyses was defined as Benjamini-Hochberg adjusted P < 0.05.

**References**

1. Marteil G, Richard-Parpaillon L, Kubiak JZ: Role of oocyte quality in meiotic maturation and embryonic development. *Reprod Biol* 2009, 9:203-224.
2. Khosla S, Dean W, Brown D, Reik W, Feil R: Culture of preimplantation mouse embryos affects fetal development and the expression of imprinted genes. *Biol Reprod* 2001, 64:918-926.
3. Fernandes G, Dasai N, Kozlova N, Mojadadi A, Gall M, Drew E, Barratt E, Madamidola OA, Brown SG, Milne AM, et al: A spontaneous increase in intracellular Ca2+ in metaphase II human oocytes in vitro can be prevented by drugs targeting ATP-sensitive K+ channels. *Hum Reprod* 2016, 31:287-297.
