# Supplementary material for: NADH supplementation improves human oocyte maturation and developmental competence of resulting embryos in controlled ovarian hyperstimulation cycles: a pilot study implicating the CDK2/GAS6 signaling pathway
Source: Front Endocrinol (Lausanne). 2025 Sep 3;16:1627679. doi: 10.3389/fendo.2025.1627679 (PMC12440754; doi:10.3389/fendo.2025.1627679)
Supplement: Supplementary Table 1 — Baseline level of female patients in each group. BMI, body mass index; FSH, follicle-stimulating hormone; E2, estrogenic hormone; P, pregestational hormone; PRL, prolactin; LH, luteinizing hormone; T, testosterone. All data are expressed as mean ± S. [file DataSheet2.zip › Appendix/Figure S1.docx]

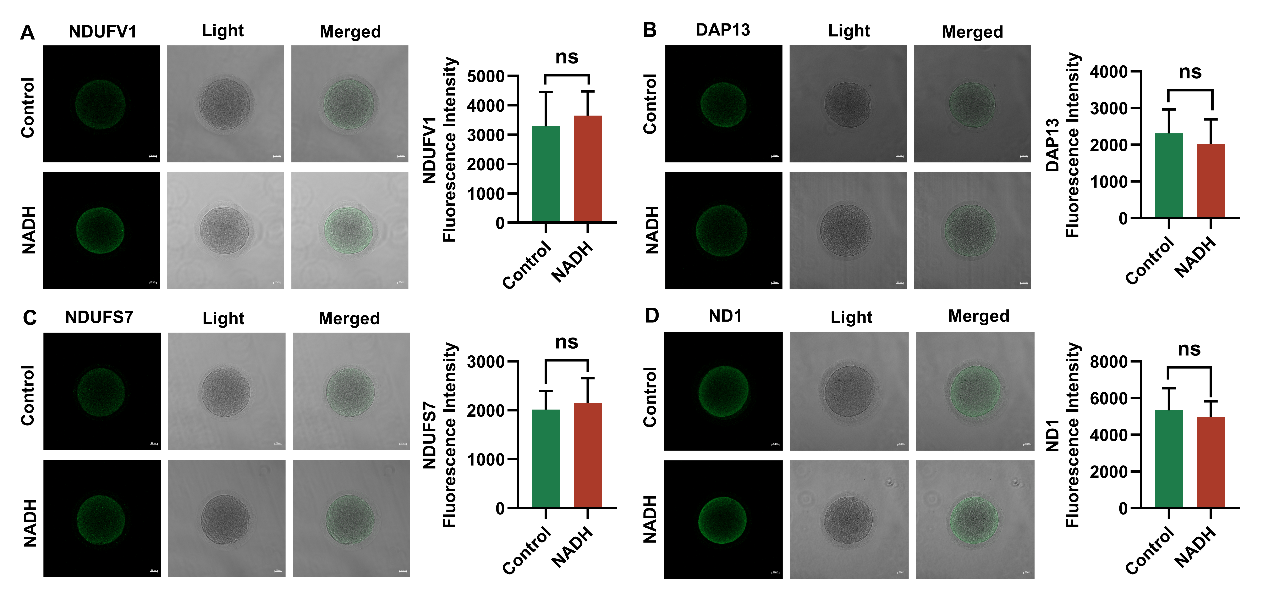


Figure S1. Fluorescence images of human IVM-MⅡ oocytes by laser confocal microscopy and statistical results of repetitive laser confocal assay experiments on the effect of 10^-6^ M NADH. Scale bar, 20 μm (A) Effect of NADH on NDUFV1 protein expression in human IVM-MⅡ oocytes. Sample size: Control group: n = 14; NADH group: n = 13. (B) Effect of NADH on DAP13 protein expression in human IVM-MⅡ oocytes. Sample size: Control group: n = 12; NADH group: n = 13. (C) Effect of NADH on NDUFS7 protein expression in human IVM-MⅡ oocytes. Sample size: Control group: n = 10; NADH group: n = 11. (D) Effect of NADH on DAP13 protein expression in human IVM-MⅡ oocytes. Sample size: Control group: n = 10; NADH group: n = 10.
