# Supplementary material for: NADH supplementation improves human oocyte maturation and developmental competence of resulting embryos in controlled ovarian hyperstimulation cycles: a pilot study implicating the CDK2/GAS6 signaling pathway
Source: Front Endocrinol (Lausanne). 2025 Sep 3;16:1627679. doi: 10.3389/fendo.2025.1627679 (PMC12440754; doi:10.3389/fendo.2025.1627679)
Supplement: Supplementary Table 1 — Baseline level of female patients in each group. BMI, body mass index; FSH, follicle-stimulating hormone; E2, estrogenic hormone; P, pregestational hormone; PRL, prolactin; LH, luteinizing hormone; T, testosterone. All data are expressed as mean ± S. [file DataSheet2.zip › Appendix/Table S1.docx]

| **Index** | **0 M (n=39)** | **10^-4^ M (n=38)** | **10^-5^ M (n=38)** | | **10^-6^ M (n=36)** | **10^-7^ M (n=36)** | **10^-8^ M (n=36)** | **Adjusted*P*-value** |
| --- | --- | --- | --- | --- | --- | --- | --- | --- |
| **Female age** | 29.44±3.51 | 29.47±3.45 | 30.21±3.50 | 30.06±4.93 | | 29.61±2.90 | 29.75±2.70 | ns |
| **BMI (kg/m^2^)** | 22.46±3.40 | 22.59±2.78 | 22.79±3.44 | 21.99±2.70 | | 23.46±3.20 | 23.72±3.59 | ns |
| **FSH (mIU/mL)** | 6.43±1.27 | 6.70±1.65 | 6.41±1.31 | 6.52±1.26 | | 7.19±3.51 | 6.50±1.67 | ns |
| **E_2_ (pM)** | 160.8±86.85 | 161.0±93.05 | 165.8±69.46 | 160.7±84.16 | | 143.8±85.06 | 148.7±85.16 | ns |
| **P (nM)** | 2.01±1.17 | 2.12±1.58 | 2.35±1.79 | 2.03±1.61 | | 2.49±1.72 | 2.43±1.71 | ns |
| **PRL (ng/mL)** | 23.79±38.09 | 18.56±13.31 | 17.24±10.76 | 17.49±12.46 | | 19.77±10.99 | 24.78±28.51 | ns |
| **LH (mIU/mL)** | 4.88±1.68 | 5.19±2.75 | 5.02±2.09 | 5.27±2.27 | | 4.39±1.91 | 4.60±2.24 | ns |
| **T (nM)** | 1.28±0.74 | 1.22±0.74 | 1.48±0.76 | 1.36±0.73 | | 1.91±3.81 | 1.90±3.81 | ns |

**Table S1.** Baseline level of female patients in each group. Note: BMI: body mass index; FSH: follicle-stimulating hormone; E2: estrogenic hormone; P: progestational hormone; PRL: prolactin; LH: luteinizing hormone; T: testosterone. All data are expressed as mean ± S
