# Supplementary material for: NADH supplementation improves human oocyte maturation and developmental competence of resulting embryos in controlled ovarian hyperstimulation cycles: a pilot study implicating the CDK2/GAS6 signaling pathway
Source: Front Endocrinol (Lausanne). 2025 Sep 3;16:1627679. doi: 10.3389/fendo.2025.1627679 (PMC12440754; doi:10.3389/fendo.2025.1627679)
Supplement: Supplementary Table 1 — Baseline level of female patients in each group. BMI, body mass index; FSH, follicle-stimulating hormone; E2, estrogenic hormone; P, pregestational hormone; PRL, prolactin; LH, luteinizing hormone; T, testosterone. All data are expressed as mean ± S. [file DataSheet2.zip › Appendix/Table S2.docx]

| **Gene Symbol** | **Type** | **Log_2_ (NADH/Control)** | **Q value (NADH/Control)** |
| --- | --- | --- | --- |
| **FIGN** | mRNA | 1.14 | <0.001 |
| **FAM9B** | mRNA | 3.26 | <0.001 |
| **GAS6** | mRNA | 1.02 | <0.001 |
| **C14orf39** | mRNA | 1.01 | <0.001 |
| **DMC1** | mRNA | 1.15 | <0.001 |
| **CDK2** | mRNA | 1.05 | <0.001 |

**Table S2.** Differentially expressed genes associated with oocyte maturation.
