# Supplementary material for: NADH supplementation improves human oocyte maturation and developmental competence of resulting embryos in controlled ovarian hyperstimulation cycles: a pilot study implicating the CDK2/GAS6 signaling pathway
Source: Front Endocrinol (Lausanne). 2025 Sep 3;16:1627679. doi: 10.3389/fendo.2025.1627679 (PMC12440754; doi:10.3389/fendo.2025.1627679)
Supplement: Supplementary Table 1 — Baseline level of female patients in each group. BMI, body mass index; FSH, follicle-stimulating hormone; E2, estrogenic hormone; P, pregestational hormone; PRL, prolactin; LH, luteinizing hormone; T, testosterone. All data are expressed as mean ± S. [file DataSheet2.zip › Appendix/Table S3.docx]

| Index | Control | NADH | CDK2 Inh | CDK2 Inh + NADH |
| --- | --- | --- | --- | --- |
| No. of immature oocytes (GV) | 23 | 23 | 26 | 24 |
| No. of immature oocytes (MІ) | 13 | 10 | 13 | 14 |
| No. of immature oocyte (GV+MІ) | 36 | 33 | 39 | 38 |
| Rate of matured oocytes (%)-24h | 61.11% (22/36) | 72.73% (24/33) | 5.13%  (2/39) | 34.21% (13/38) |
| Rate of matured oocytes (%)-48h | 72.22% (26/36) | 87.88% (29/33) | 10.26% (4/39) | 57.89% (22/38) |

**Table S3.** Effects of different concentrations of CDK2 inhibitor on oocyte maturation. Note: Rate of maturation: the number of in-vitro matured oocytes (IVM-MⅡ)/the number of immature oocytes (GV+MІ)
