# Supplementary material for: NADH supplementation improves human oocyte maturation and developmental competence of resulting embryos in controlled ovarian hyperstimulation cycles: a pilot study implicating the CDK2/GAS6 signaling pathway
Source: Front Endocrinol (Lausanne). 2025 Sep 3;16:1627679. doi: 10.3389/fendo.2025.1627679 (PMC12440754; doi:10.3389/fendo.2025.1627679)
Supplement: Supplementary Table 1 — Baseline level of female patients in each group. BMI, body mass index; FSH, follicle-stimulating hormone; E2, estrogenic hormone; P, pregestational hormone; PRL, prolactin; LH, luteinizing hormone; T, testosterone. All data are expressed as mean ± S. [file DataSheet2.zip › Appendix/Table S4.docx]

| Index | Control | GAS6 |
| --- | --- | --- |
| Rate of immature oocyte development into blastocyst (%) | 4.35%  (2/46) | 27.27%  (12/44) |
| Rate of fertilization (%) | 88.46%  (23/26) | 93.94%  (31/33) |
| Rate of cleavage (%) | 91.30% (21/23) | 87.10% (27/31) |
| Rate of high-quality embryo (%) | 23.81%  (5/21) | 18.52% (5/27) |
| Rate of blastocyst (%) | 9.52%  (2/21) | 44.44% (12/27) |
| Rate of high-quality blastocyst (%) | 4.76%  (1/21) | 11.11% (3/27) |

Table S4. Embryoic development in the GAS6 protein supplemented group and the non-supplemented group. Note: Rate of immature oocyte development into blastocyst (%): the number of blastocysts/the number of immature oocytes. Rate of fertilization: the number of fertilized oocytes/the number of mature oocytes. Rate of cleavage: the number of cleaved embryos/the number of fertilized oocytes. Rate of high-quality embryo: the number of high-quality embryos on day 3/the number of cleaved embryos. Rate of blastocyst: the number of blastocysts/the number of cleavage embryos. Rate of high-quality blastocyst: the number of high-quality blastocysts/the number of cleavage embryos.
